# Primary Sjögren's syndrome manifesting as multiple cranial neuropathies: MRI findings

**DOI:** 10.4103/0972-2327.53083

**Published:** 2009

**Authors:** V. V. Ashraf, Ramesh Bhasi, R. Praveen Kumar, A. S. Girija

**Affiliations:** Department of Neurology, Malabar Institute of Medical Sciences, Calicut - 673 016, Kerala, India; 1 Department of Rheumatology, Malabar Institute of Medical Sciences, Calicut - 673 016, Kerala, India

**Keywords:** Cranial pachymeningitis, multiple cranial nerve palsies, primary Sjögren syndrome

## Abstract

We report a case of primary Sjögren's syndrome presenting with multiple cranial nerve palsies and radiological evidence of cranial pachymeningitis and hypophysitis. A 47-year-old woman developed right sensory neural hearing loss followed, 2 months later, by right facial palsy. Cranial magnetic resonance imaging showed features of pachymeningitis and pituitary gland infiltration. The diagnosis of primary Sjögren's syndrome was confirmed by demonstrating positive SS-A and SS-B antibodies and histological evidence of lymphocytic infiltration of the sublabial salivary gland. During the 2-year follow-up, the patient had transient VI^th^, IX^th^, X^th^, and XII^th^ cranial nerve palsies. Sjögren's syndrome should be considered in the differential diagnosis of patients presenting with multiple recurrent cranial nerve palsies, even if prominent sicca symptoms are absent.

## Introduction

Sjögren syndrome (SS) is a chronic, inflammatory, autoimmune disease characterized by lymphocytic infiltration of exocrine glands, leading to dry mouth and dry eyes and resulting in the so-called sicca complex. Peripheral nervous system disease, manifesting commonly as peripheral sensory neuropathy or, more rarely, as mononeuritis multiplex, is a well-established feature of the disease and occurs in approximately a quarter of the patients.[1] However, less frequently, cranial neuropathy can also occur, which is especially likely to involve the trigeminal nerve.[2] About 5-25% of patients with primary SS have central nervous system (CNS) manifestations, including focal brain lesions, diffuse brain lesions, and spinal cord lesions.[3] A recurrent neurological syndrome resembling multiple sclerosis also occurs. We report a patient with primary SS, multiple cranial neuropathies, and radiological evidence of pachymeningitis and pituitary gland infiltration.

## Case Report

A 47-year-old woman presented with 2 days' history of right facial weakness and pain in the right periauricular area. Two months earlier she had had acute-onset deafness on the right side. The general physical examination was unremarkable. Neurological examination showed right lower motor neuron facial palsy and reduced taste sensation on the right half of the tongue. Hearing was reduced on the right side and the Rinne test and Weber test were suggestive of sensory neural hearing loss (SNHL).

**Figure 1:**
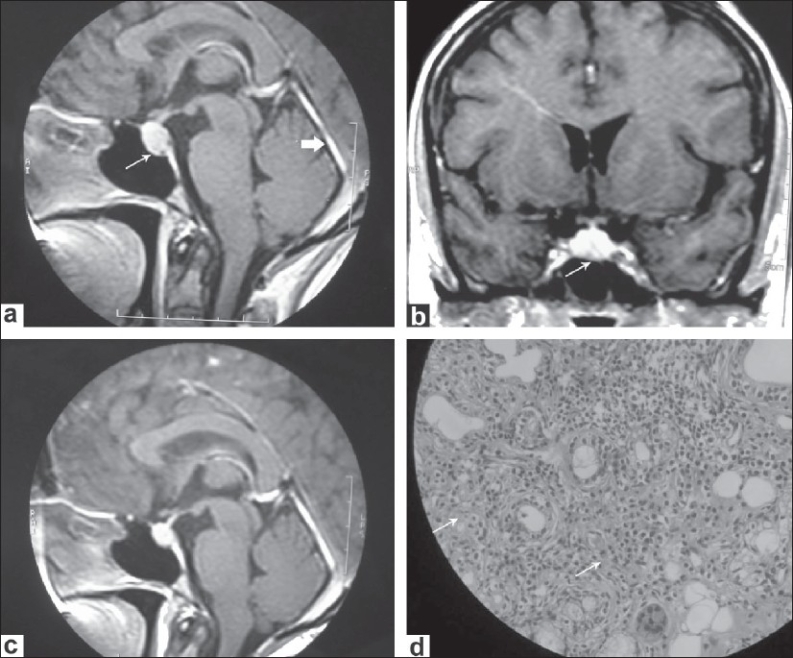
(a) Sagittal gadolinium-enhanced T1-weighted MR images showing thickening and enhancement of cerebellar tentorium (short thick arrow). It also shows a mildly enlarged pituitary gland. (b) Coronal gadolinium-enhanced T1-weighted MR images showing the enlarged and enhancing pituitary gland. (c) Sagittal T1-weighted MR image (post contrast) showing reduction in size of pituitary gland swelling and dural thickening following immunotherapy. (d) Histopathology section of lower lip biopsy showing diffuse infiltration of lymphocytes and plasma cells

Although the erythrocyte sedimentation rate (ESR) was 105 mm/h, other routine laboratory investigations were normal. Magnetic resonance imaging (MRI) of the brain showed mild diffuse thickening and gadolinium enhancement of the duramater in the falx cerebri and cerebellar tentorium. Furthermore, an intensely enhancing, enlarged pituitary gland was also noted [Figures 1a and b]. An audiogram revealed moderate right SNHL.

Review of the patient's medical history showed that she had had complaints of a dry sensation of the mouth, burning eyes, and polyarthralgias for the past 2 years. Rheumatoid factor was positive at a titer of 53 IU/ml (normal: <15) and antinuclear antibody (ANA) was positive at a titer of 1.10 by the enzyme immunoassay technique (normal: <0.7). Antibodies to SS-A and SS-B were also strongly positive. Anti-ds DNA and antibodies to Sm, RNP, and Scl-70 were negative. Serum immune electrophoresis revealed no monoclonal gammopathy. Schirmer test showed decreased lacrimation and a lower labial biopsy showed significant lymphocytic infiltration [Figure 1d]; these findings were consistent with the diagnosis of Sjögren syndrome (SS). Nerve conduction study revealed bilateral mild carpal tunnel syndrome and right facial neuropathy. Additionally, a cerebrospinal fluid (CSF) study showed normal protein and sugar and a cell count of 5 cells/mm^3^ (all lymphocytes). Gram's staining and AFB staining of the CSF was normal and the VDRL test was negative. Serum angiotensin converting enzyme (ACE) level was also normal. In view of the pituitary gland infiltration, an endocrine workup was done. Thyroid function test, serum cortisol and ACTH levels, and the water deprivation test for diabetes insipidus were all normal; serum prolactin was mildly raised at 37.6 ng/ml (normal: 2-25 ng/ml). Based on these findings, we diagnosed primary SS, with probable lymphocytic CNS involvement. The patient was treated with IV methylprednisolone pulse therapy, with 1 g/day for three successive days, followed by 0.5 mg/kg/day oral prednisolone, with gradual tapering of the dose. Later, weekly methotrexate at a dose of 10 mg/week was added. Follow-up brain MRI after 6 months of treatment showed a minimal decrease in the pituitary gland enlargement and the meningeal thickening [Figure 1c]. The patient's symptoms, except for the dryness of the mouth, resolved with treatment. Over the next 1 year, she had three episodes of transient diplopia due to lateral rectus palsy. She recovered from all these attacks within a few weeks after the dose of steroids was increased. Two years after the first evaluation, she was readmitted with acute-onset dysphagia, nasal regurgitation, and hoarseness of voice. On examination, she was found to have right IX^th^, X^th^, and XII^th^ cranial nerve palsies, without any long tract signs or cerebellar signs. Repeat MRI did not reveal any fresh brainstem lesions. She was treated with a 3-day course of methylprednisolone injection (1 g/day) and recovered completely in 2 weeks' time.

## Discussion

SS is a chronic inflammatory disorder of the exocrine glands, which primarily affects middle-aged women. Diagnosis of SS often depends on the recognition of subtle manifestations of the sicca complex. In some patients (such as ours), neurological manifestations can predate or overshadow symptomatic glandular involvement. Laboratory evaluations are indispensable in these situations. Multiple serological abnormalities are present in most patients with SS. Rheumatoid factor is present in 75-90% of SS patients even in the absence of arthritis.[3] Positive antinuclear antibody (ANA) occurs in nearly 70% of SS patients. Antibodies to the nucleoprotein antigen SS-B (La) occurs in 50-70% of patients with primary SS. Anti-SS-A (R0) is less sensitive, but has better prognostic value in patients with CNS disease.[4]

Cranial neuropathy is an infrequent manifestation of SS. Any cranial nerve from II to XII can be affected, with trigeminal sensory neuropathy being the commonest.[2,3,5] Tonic pupils with light-near dissociation have been described in patients with peripheral neuropathy due to SS (Adie pupil).[6] This may be an isolated occurrence or part of generalized autonomic neuropathy. Trigeminal neuropathy may represent a local ganglionitis affecting the gasserian ganglion and may be a limited form of pure sensory neuronopathy. The mechanism of the other cranial neuropathies is believed to be vasculitic damage of the vasa nervosum.[2] Our patient had VI^th^, VII^th^, VIII^th^, IX^th^, X^th^, and XII^th^ cranial nerve involvement. Only very few cases of facial, abducent, and lower cranial nerve palsies have been described previously in primary SS.[5,7] SNHL is reported in nearly 27% of patients of SS, although clinically significant defects are not common.[8]

Two other interesting radiological features in this patient are the presence of pachymeningitis and MRI evidence of pituitary gland infiltration, though our patient had no clinical evidence of hypopituitarism or central diabetes insipidus. Lymphocytic infiltration of the pituitary gland has been reported in SS as causing a secondary form of lymphocytic hypophysitis.[9] Sometimes this can be asymptomatic, as in our patient. The imaging features in our patient are more in favor of hypophysitis than a pituitary adenoma. The MRI findings are not specific enough to distinguish hypophysitis from the more common pituitary adenomas with certainty. However, the symmetry of pituitary enhancement, the lack of erosive changes of the sellar floor, and the homogeneity of the pituitary mass and its intense enhancement after gadolinium can be diagnostic in the proper clinical setting.[10] Cranial pachymeningitis is an extremely rare manifestation of primary SS.[11] Pachymeningitis is characterized by dural thickening, which typically involves the falx cerebri and the tentorium. The most common symptoms include headache, ataxia, and cranial nerve palsies. Common etiologies of cranial pachymeningitis include sarcoidosis, Wegener granulomatosis, neurosyphilis, tuberculosis, and lymphoma; SS is an extremely rare cause for this.[11]

Most of the systemic complications of SS have been explained as being either due to vasculitis or due to an extension of the inflammatory lymphoproliferative process to extraglandular locations. The pathogenesis of CNS involvement could be categorized into two distinct forms: Vasculitis and polymorphous meningitis.[12] The latter may represent leptomeningeal involvement in the inflammatory lymphoproliferative process of SS, whereas the former is probably immune mediated.

This case illustrates how facial palsy and deafness led to the realization that primary SS was the underlying systemic disorder. To the best of our knowledge, the combination of facial palsy, sensory neural deafness, and pachymeningitis as the presenting manifestation of primary SS has not been reported hitherto.
